# Asparaginase induces apoptosis and cytoprotective autophagy in chronic myeloid leukemia cells

**DOI:** 10.18632/oncotarget.2869

**Published:** 2015-01-08

**Authors:** Ping Song, Li Ye, Jiajun Fan, Yubin Li, Xian Zeng, Ziyu Wang, Shaofei Wang, Guoping Zhang, Ping Yang, Zhonglian Cao, Dianwen Ju

**Affiliations:** ^1^ Department of Biosynthesis & The Key Laboratory of Smart Drug Delivery, Ministry of Education, School of Pharmacy, Fudan University, Shanghai, 201203, P.R. China; ^2^ Institute of Biomedical Science, Fudan University, Shanghai, 200032, P.R. China; ^3^ Instrumental Analysis Center, School of Pharmacy, Fudan University, Shanghai, 201203, P.R. China

**Keywords:** asparaginase, autophagy, apoptosis, chronic myeloid leukemia

## Abstract

The antitumor enzyme asparaginase, which targets essential amino acid L-asparagine and catalyzes it to L-aspartic acid and ammonia, has been used for years in the treatment of acute lymphoblastic leukemia (ALL), subtypes of myeloid leukemia and T-cell lymphomas, whereas the anti-chronic myeloid leukemia (CML) effect of asparaginase and its underlying mechanism has not been completely elucidated. We have shown here that asparaginase induced significant growth inhibition and apoptosis in K562 and KU812 cells. Apart from induction of apoptosis, we reported for the first time that asparaginase induced autophagic response in K562 and KU812 cells as evidenced by the formation of autophagosome, microtubule-associated protein light chain 3 (LC3)-positive autophagy-like vacuoles, and the upregulation of LC3-II. Further study suggested that the Akt/mTOR (mammalian target of rapamycin) and Erk (extracellular signal-regulated kinase) signaling pathway were involved in asparaginase-induced autophagy in K562 cells. Moreover, blocking autophagy using pharmacological inhibitors LY294002, chloroquine (CQ) and quinacrine (QN) enhanced asparaginase-induced cell death and apoptosis, indicating the cytoprotective role of autophagy in asparaginase-treated K562 and KU812 cells. Together, these findings provide a rationale that combination of asparaginase anticancer activity and autophagic inhibition might be a promising new therapeutic strategy for CML.

## INTRODUCTION

CML is a myeloproliferative neoplasm with an incidence of 1–2 cases per 100,000 adults, and accounts for ~15% of newly diagnosed cases of leukemia in adults. A significant percentage of the patients with CML failed to respond effectively to the current regimen of drug therapy including frontline tyrosine kinase inhibitors (TKIs) therapy, and had to be considered for allogeneic stem cell transplantation (AlloSCT) which has a high risk of morbidity and mortality [1–3]. The prevalence of CML represents a considerable burden on patients and the healthcare systems in regard to drug availability, potential development of long-term side effects, and costs [4, 5]. Therefore, it is critical to continue research into novel therapeutic approaches.

Targeting amino acid metabolism has been safely and effectively employed for tumor therapy [6]. Asparaginase, a Food and Drug Administration (FDA)-approved enzyme therapeutics for cancer therapy, has been used to treat ALL since the early 1970s and induces a 60% of complete remission (CR) rate as a monotherapy [7]. Tumor cells, more specifically leukemia cells, require huge amounts of asparagine to keep up with their rapid malignant growth. Therefore L-asparagine is an essential amino acid for the growth of tumor cells, whereas the growth of normal cells is not dependent on its requirement as it can be synthesized in amounts sufficient for their metabolic needs with their own enzyme L-asparagine synthetase (ASNS) [8, 9]. The presence of therapeutic asparaginase deprives tumor cells of an important growth factor by hydrolyzing L-asparagine into L-aspartic acid and ammonia, afterwards tumor cells fail to survive because of their reduced ASNS levels [10]. Asparaginase could also deprive L-glutamine, which is a precursor of L-asparagine, thereby producing L-glutamic acid and ammonia [10]. Although primarily used as a chemotherapeutic agent against ALL [11, 12], asparaginase is also used in other types of leukemia such as non-Hodgkin's lymphoma [13], subtypes of myelocytic leukemia [14] and chronic lymphocytic leukemia, sarcomas such as lymphosarcoma, reticulosarcoma and melanosarcoma [15], ovarian cancer [16] and brain cancer [6] with a potential role for its glutaminase activity [10].

One of the key cellular responses to nutrient withdrawal is the upregulation of autophagy [17], and mounting evidence suggest that amino-acid depletion could concurrently induce autophagy and apoptosis [18–21]. Autophagy is a cellular catabolic process that contributes to quality control and maintenance of the cellular energetic balance through the turnover of proteins and organelles in lysosomes, and takes place at basal levels in most of the cell types but is also regulated by environmental stimuli [22]. In fact, autophagy is a process by which cells can adapt their metabolism to starvation caused by a decrease in metabolite concentrations or extracellular nutrients allowing cells to evade programmed cell death [23]. Accordingly, inhibition of autophagy results in cell death of growth factor-starved cells [24]. In tumors displaying defective apoptosis, inhibition of autophagy causes caspase-independent necrotic cell death, which, in turn, augments inflammation, leading to enhanced tumor burden [25, 26].

Recent study showed that L-asparaginase inhibited mTORC1, and induced apoptosis as well as autophagic process in acute myeloid leukemia (AML) cells [14]. Autophagy was also observed in ovarian cancer cell exposed to asparaginase at physiologically attainable concentrations with induction of ATG12, beclin-1, and cleavage of LC3 [27]. It has been reported that autophagy plays an important role in CML tumourgenesis, progression and therapy [28]. Imatinib mesylate (IM), a TKI as the first-line therapy for patients with CML, could induce autophagy in CML cells, and autophagy inhibitors enhanced the therapeutic effects of TKIs in the treatment of CML [28, 29]. Despite of these advances, there has been few investigation on targeting asparagine metabolism in CML therapy. Whether asparaginase could induce autophagy and apoptosis, and the relationship between them in CML cells remain unknown.

In this study, we report that asparaginase induces obvious growth inhibition and apoptosis in CML cells. Meanwhile, apoptosis is not the sole consequence of asparagine deprivation, as asparaginase treatment rapidly activates an autophagic process by inducing the conversion of LC3-I to LC3-II. In addition, the Akt/mTOR (mammalian target of rapamycin) and Erk (extracellular signal-regulated kinase) signaling pathway are involved in asparaginase-induced autophagy in K562 cells. Of greater importance, inhibition of autophagy by pharmacological inhibitors enhances asparaginase-induced cell death in CML cells. These findings indicate that autophagy provides a cytoprotective mechanism in CML cells treated by asparaginase, and inhibition of autophagy may improve the therapeutic efficacy of asparaginase in the treatment of CML. Taken together, these results suggest that combination of asparaginase anticancer activity and autophagic inhibition might be a promising new therapeutic strategy for CML.

## RESULTS

### Asparaginase induces growth inhibition and apoptosis in K562 and KU812 CML cells

**Figure 1:**
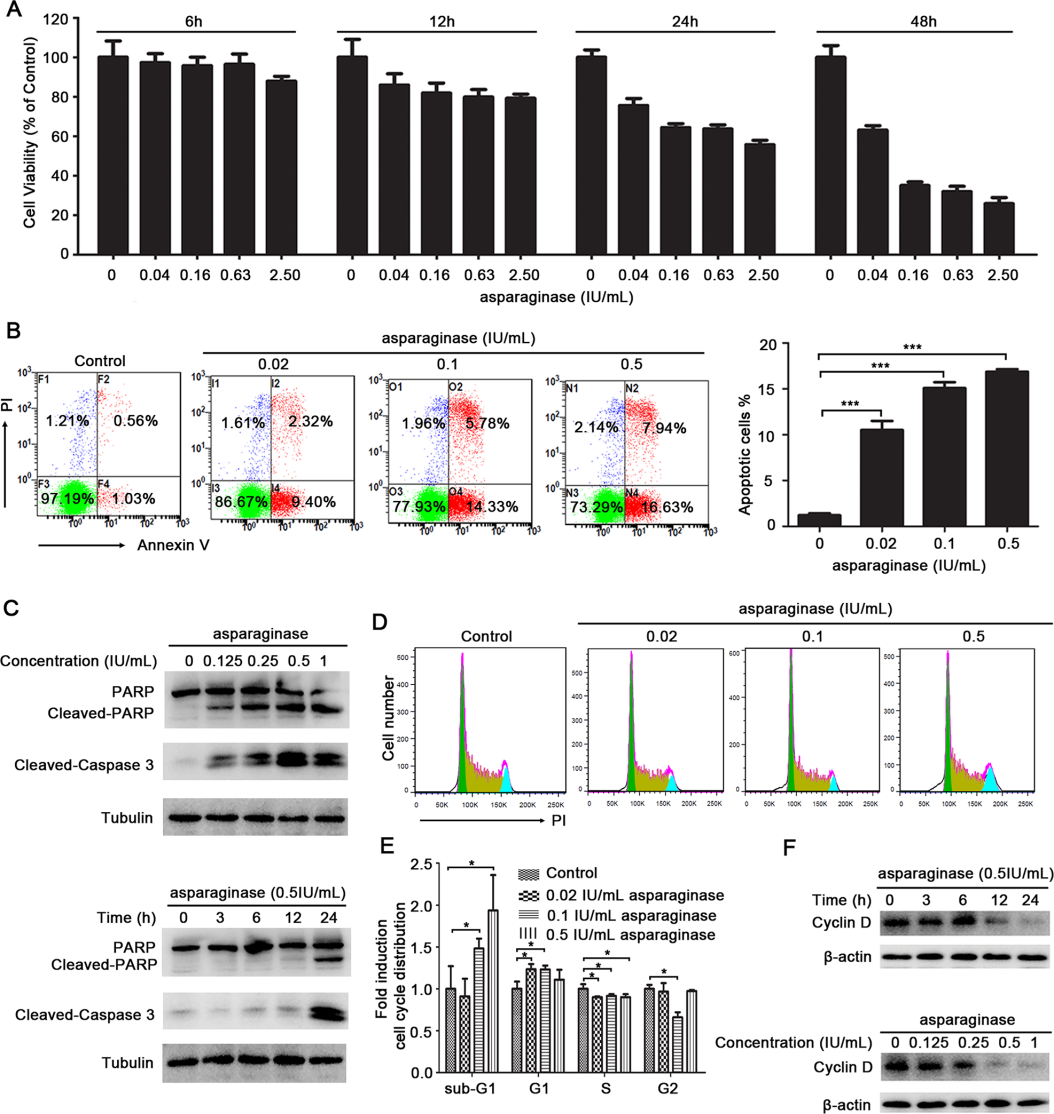
Asparaginase induces growth inhibition and apoptosis in K562 CML cells **(A)** K562 cells were incubated with different concentrations of asparaginase for 6, 12, 24, and 48 h, then cell viability was measured by MTT assay. **(B)** K562 cells were treated with 0.02, 0.1, 0.5 IU/mL of asparaginase for 48 h, and stained with Annexin V/PI, then analyzed by flow cytometry. The percentages of Annexin V-positive/PI-negative cells were presented in bar charts. **(C)** K562 cells were dose- and time-dependently treated with asparaginase, then western blot analysis was performed to assess the expression level of cleaved-caspase 3, PARP and cleaved-PARP. **(D)** K562 cells were treated with 0.02, 0.1, 0.5 IU/mL of asparaginase for 24 h, cell cycle distribution were analyzed by flow cytometry. **(E)** Quantification of cells in different phases were normalized to control and presented in bar graphs. (F) K562 cells were dose- and time-dependently treated with asparaginase, the protein cyclin D was analyzed by western blot analysis. Results were represented as mean ± SD (*P < 0.05, ***P < 0.001).

Firstly, we determined the growth inhibitory effect of asparaginase in K562 and KU812 cells. As shown in Figure 1A and Supplementary Figure 1A, asparaginase reduced cell viability in a dose- and time-dependent manner. In addition, treatment of K562 and KU812 cells with different concentrations of asparaginase for 48 h increased the percentage of apoptotic cells (Figure 1B and Supplementary Figure 1B, 1C). Meanwhile, western blot analysis illustrated that the level of cleaved-caspase 3 and cleaved-PARP increased in a dose- and time-dependent manner, indicating the apoptosis was induced by asparaginase in K562 and KU812 cells (Figure 1C and Supplementary Figure 1D).

Secondly, the effect of asparaginase in K562 cell cycle distribution was performed by FACS analysis after stained with PI. As shown in Figure 1D and 1E, the cells at sub-G1 phase in these asparaginase-treated groups significantly increased when compared with negative controls, indicating that asparaginase could induce cell death in K562 cells. In addition, upon the asparaginase treatment, the cells at G1 phase increased with reduced cells at S phase when compared with negative controls, indicating that asparaginase could induce G1 arrest to decelerate the cell cycle, and prevent the cells from entering the S phase and proliferating. Furthermore, western blot analysis revealed a gradual reduction of Cyclin D in a time- and dose-dependent manner in K562 cells after asparaginase treatment (Figure 1F). Cyclin D is a cell cycle regulator essential for G1 phase, and expression of Cyclin D correlate closely with development and prognosis of cancers [30, 31]. Thus, reduction of Cyclin D indicates cell cycle arrest and cell growth inhibition.

These results demonstrate that asparaginase induces growth inhibition and apoptosis in K562 and KU812 CML cells.

### Asparaginase-induced apoptosis is partially caspase 3-dependent in K562 CML cells

**Figure 2:**
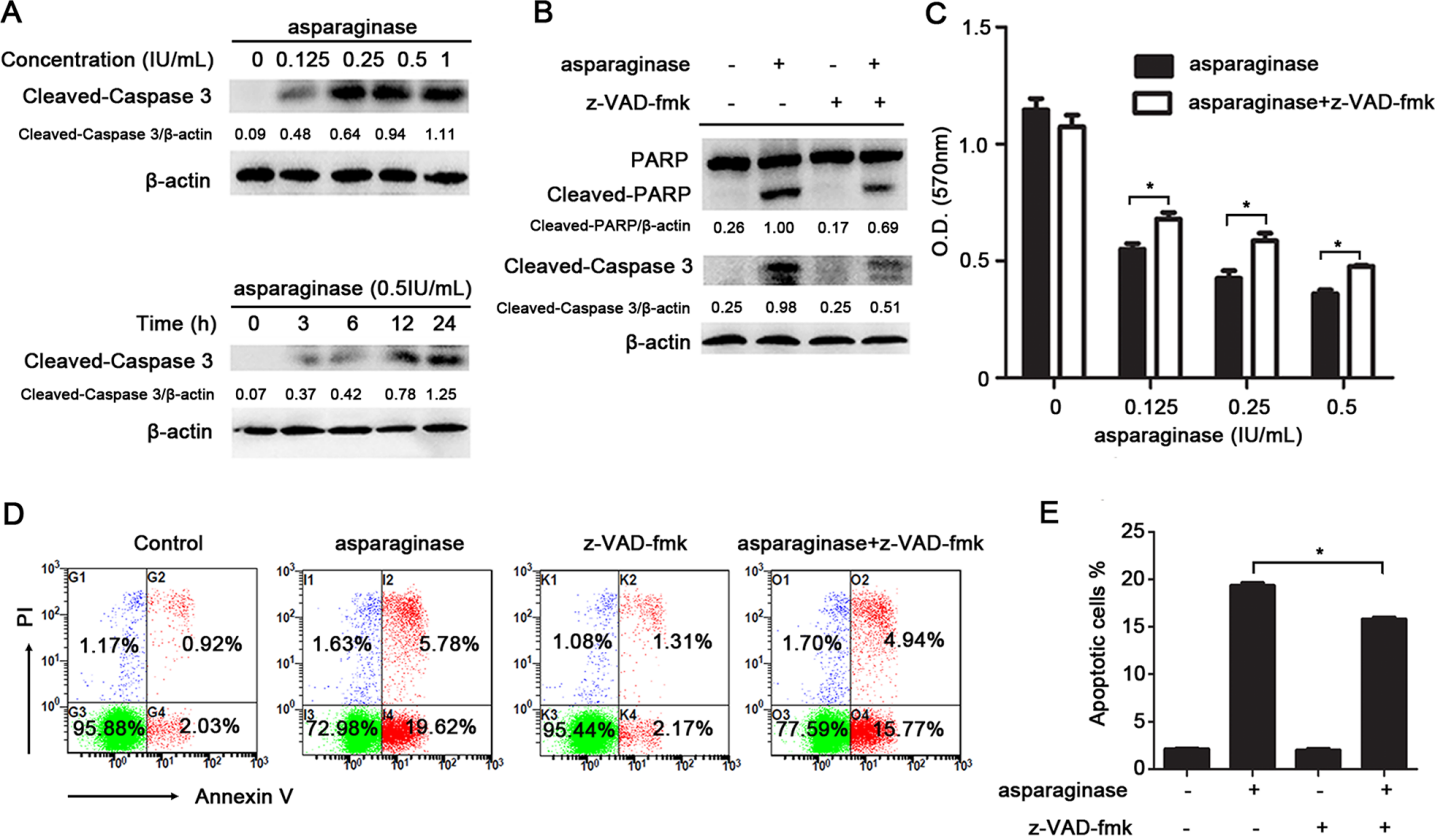
Apoptosis induced by asparaginase is partially caspase 3-dependent in K562 CML cells **(A)** K562 cells were dose- and time-dependently incubated with asparaginase, then western blot analysis was performed to assess the level of cleaved-caspase 3. Densitometric values were quantified using the ImageJ software, and the data represented mean of three independent experiments. **(B)** K562 cells were incubated with 0.5 IU/mL of asparaginase, either alone or in combination with 20 μM z-VAD-fmk for 24 h, then western blot analysis was performed to assess the level of cleaved-caspase 3, PARP and cleaved-PARP. Densitometric values were quantified using the ImageJ software, and the data are presented as means ± SD of three independent experiments. **(C–E)** K562 cells were treated with asparaginase at indicated concentrations in the absence or presence of 20 μM z-VAD-fmk for 48 h. **(C)** Cell viability was determined by MTT assay at the wavelength of 570 nm. **(D)** Cells were stained with Annexin V/PI and analyzed by flow cytometry after 48 h incubation. **(E)** The percentages of Annexin V-positive/PI-negative cells were presented in bar charts. Results were represented as mean ± SD (*P < 0.05).

K562 cells were exposed to asparaginase for the measurement of apoptosis. The western blot analysis showed that treatment with asparaginase dramatically induced the cleavage of caspase 3 in K562 cells in both a dose- and time-dependent manner (Figure 2A). To further demonstrate whether asparaginase-induced apoptosis in K562 cells was correlated to the activation of caspase 3, a pan-caspase inhibitor benzyloxycarbonyl Val-Ala-Asp (O-methyl)-fluoro-methylketone (z-VAD-fmk) was employed. The results showed that 20 μM of z-VAD-fmk could significantly decrease the level of cleaved-caspase 3 (Figure 2B). In addition, when asparaginase was combined with the treatment of z-VAD-fmk, the level of cleaved-PARP (Figure 2B), the percentage of growth inhibition (Figure 2C) and apoptotic cells (Figure 2D and Figure 2E) were significantly decreased.

These results reveal that asparaginase-induced apoptosis in K562 CML cells partially depends on caspase 3 activation.

### Asparaginase induces autophagy in K562 and KU812 CML cells

**Figure 3:**
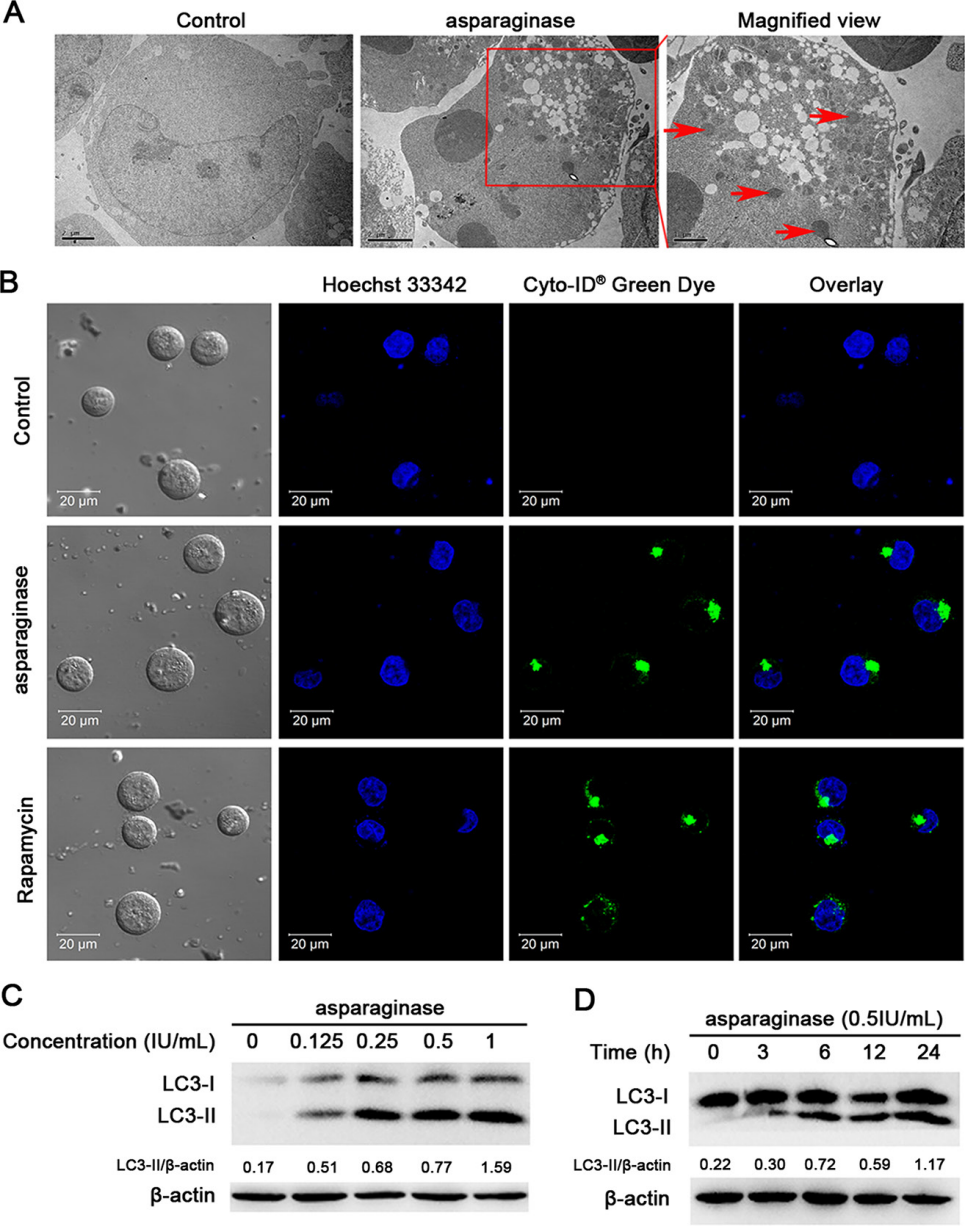
Autophagy is induced by asparaginase in K562 cells **(A)** K562 cells were treated with 0.5 IU/mL of asparaginase for 24 h. TEM was employed to detect the autophagosomes (“red arrows”: autophagosomes). **(B)** K562 cells were treated with 0.5 IU/mL of asparaginase for 24 h, then cells were stained with Cyto-ID® Green autophagy dye and examined by confocal fluorescent microscopy. 50 nM of Rapamycin was regarded as positive control. **(C)** K562 cells were treated with 0.125, 0.25, 0.5 and 1 IU/mL of asparaginase for 24 h, then detected autophagy-associate protein LC3-I/II by western blot analysis. Densitometric values were quantified using the ImageJ software, and the data represented mean of three independent experiments. **(D)** K562 cells were treated with 0.5 IU/mL of asparaginase for 3, 6, 12 and 24 h, the expression level of LC3-I/II were evaluated by western blot analysis. Densitometric values were quantified using the ImageJ software, and the data are presented as means ± SD of three independent experiments.

Previous studies have demonstrated that amino-acid depletion could induce autophagy [18]. To determine whether asparaginase induced autophagy in K562 and KU812 cells, three well-established methods were used to detect autophagosome formation. First of all, we investigated the number of autophagic vacuoles presenting in cells through transmission electron microscopy (TEM) analysis. Increasing accumulation of double-membrane-enclosed autophagosome was observed in cells after 24 h-asparaginase treatment, whereas no autophagosome was found in untreated control cells (Figure 3A and Supplementary Figure 2A). Next, we used a Cyto-ID Green dye autophagy detection kit to detect LC3-II, the protein bound on the membrane of autophagosomes with fluorescence microscopy. After treatment with 0.5 IU/mL asparaginase for 24 h, K562 and KU812 cells displayed more green fluorescence than that in the negative controls which showed limited specific fluorescence. Meanwhile, the positive controls, cells treated with 50 nM Rapamycin, exhibited significant green fluorescence (Figure 3B and Supplementary Figure 2B). Finally, we examined the conversion of LC3, also known as ATG8, to assess autophagy levels in asparaginase-treated K562 and KU812 cells through western blot analysis. Autophagosome formation is invariably associated with conversion of LC3 from the cytosolic LC3-I to the autophagosome-associated LC3-II form. Figure 3C and Supplementary Figure 2C showed the appearance of LC3-II in the cells treated with 0.125 IU/mL of asparaginase, and an obvious conversion of endogenous LC3-I to LC3-II in a dose-dependent manner. Moreover, Figure 3D and Supplementary Figure 2D revealed that the accumulation of LC3-II in protein extracts of 0.5 IU/mL asparaginase treated cells gradually increased with the extension of time, indicating autophagosome formation.

These observations strongly suggest that autophagy is induced in K562 and KU812 CML cells after asparaginase treatment.

### Blocking autophagy enhances asparaginase-induced growth inhibition and apoptosis of K562 and KU812 CML cells

**Figure 4:**
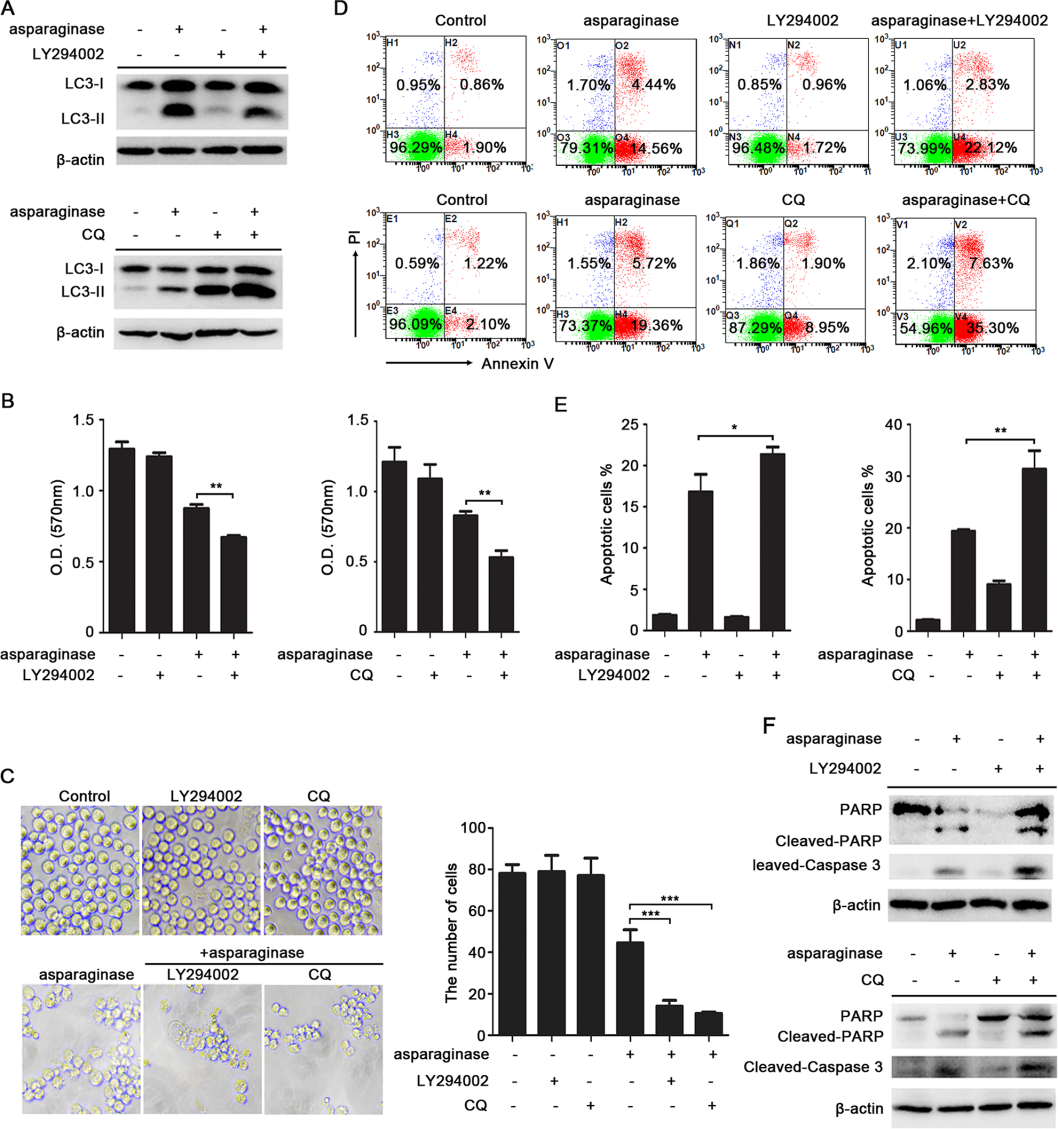
Inhibition of autophagy enhances asparaginase-induced K562 cell death **(A)** K562 cells were treated with 0.04 IU/mL of asparaginase in the absence or presence of 20 μM LY294002 or 10 μM CQ for 24 h, autophagy-associated protein LC3-I/II were detected by western blot analysis. **(B–E)** K562 cells were incubated with 0.04 IU/mL of asparaginase in the absence or presence of 20 μM LY294002 or 10 μM CQ for 48 h. **(B)** Cell viability was analyzed by MTT assay. **(C)** Morphological and numerary changes of K562 cells were observed using microscopy and photography. The number of normal cells was presented in bar charts. **(D)** Cell apoptosis was detected by Annexin V-FITC/PI staining. **(E)** The percentage of Annexin V-positive/PI-negative K562 cells was presented in bar charts. **(F)** K562 cells were treated with 0.04 IU/mL of asparaginase in combination with or without 20 μM LY294002 or 10 μM CQ for 24 h, the expression level of protein cleaved-caspase 3, PARP and cleaved-PARP were analyzed by western blot analysis. Results were represented as mean ± SD (*P < 0.05, **P < 0.01, ***P < 0.001).

Several studies have suggested that autophagy may act as a protective mechanism in tumor cells and that therapy-induced cell death can be enhanced upon autophagy inhibition [24, 32, 33]. To test whether autophagy acts as a cytoprotective mechanism in our system, we inhibited autophagy in CML cells using LY294002, chloroquine (CQ) and quinacrine (QN) [34, 35], and analyzed the effects on the level of LC3-II and asparaginase-induced cell death. LY294002 is an inhibitor of PI3K, which inhibits autophagosomes accumulation and inhibits the conversion of LC3-I to LC3-II. However CQ and QN, two lysosome inhibitors, could lead to the aggregation of autophagosomes and increase LC3-II level by blocking the fusion of autophagosomes and lysosomes. Western blot analysis indicated that asparaginase-induced autophagy was successfully inhibited by LY294002, CQ and QN (Figure 4A and Supplementary Figures 3A, 4A).

Compared with K562 and KU812 cells that incubated with asparaginase, treatment with LY294002, CQ or QN significantly increased asparaginase-induced cytotoxicity in K562 and KU812 cells (Figure 4B and Supplementary Figures 3B, 4B). Direct observations through microscope showed that asparaginase in combination with LY294002, CQ or QN induced more obvious morphology changes including cell shrinkage, fragmentation, and death when compared with asparaginase-treated alone (Figure 4C and Supplementary Figures 3C, 4C). To further understand the biological role of autophagy in asparaginase-induced cell death, we examined the changes of asparaginase-induced apoptosis. The results demonstrated that asparaginase in combination with LY294002, CQ or QN induced a higher percentage of apoptotic cells (Figure 4D, 4E and Supplementary Figures 3D, 3E, 4D, 4E) and more cleavage of caspase 3 and PARP (Figure 4F and Supplementary Figures 3F, 4F) when compared with asparaginase-treated alone, whereas cells treatment with LY294002, CQ and QN alone showed limited apoptosis-inducing effects on K562 and KU812 cells.

These results reveal that inhibition of autophagy enhances asparaginase-induced growth inhibition, morphology changes and apoptosis, indicating that autophagy plays a cytoprotective role in asparaginase-induced cell death in K562 and KU812 CML cells.

### The Akt/mTOR and Erk signaling pathway are involved in autophagy induced by asparaginase in K562 CML cells

**Figure 5:**
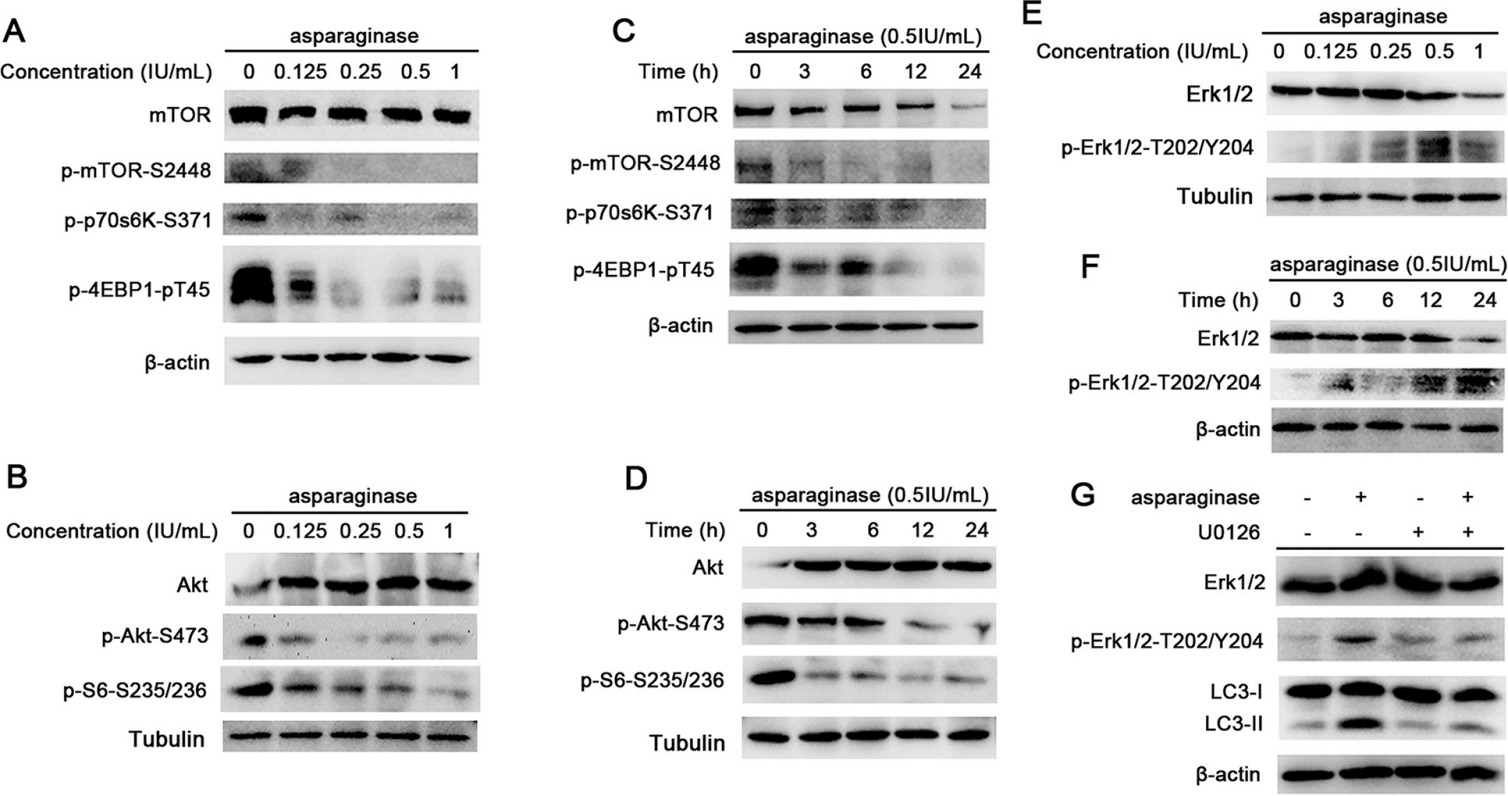
Both Akt/mTOR and Erk signaling pathway are involved in asparaginase-induced autophagy in K562 cells **(A)** K562 cells were treated with different concentrations of asparaginase for 24 h, the level of mTOR, p-mTOR, p-P70S6K and p-4EBP1 were analyzed by western blot. **(B)** K562 cells were incubated with different concentrations of asparaginase for 24 h, then western blot was performed to analyze the protein Akt, p-Akt and p-S6. **(C)** K562 cells were treated with 0.5 IU/mL of asparaginase for 3, 6, 12, 24 h, then western blot was performed to analyze the protein mTOR, p-mTOR, p-P70S6K and p-4EBP1. **(D)** K562 cells were incubated with 0.5 IU/mL of asparaginase for 3, 6, 12, 24 h, the expression level of Akt, p-Akt and p-S6 were analyzed by western blot. **(E)** K562 cells were treated with different concentrations of asparaginase for 24 h. the level of Erk 1/2 and p-Erk 1/2 were analyzed by Western blot. **(F)** K562 cells were treated with 0.5 IU/mL of asparaginase for 3, 6, 12, 24 h, then western blot was performed to analyzed the protein Erk 1/2 and p-Erk1/2. **(G)** K562 cells were incubated with 0.5 IU/mL of asparaginase in the presence or absence of the Erk phosphorylation inhibitor U0126 (20 μM) for 24 h. The level of LC3-I/II, Erk 1/2 and p-Erk 1/2 was determined by western blot analysis.

The Akt/mTOR signaling pathway is one of the major pathways regulating autophagy in eukaryotic cells. Nutrient starvation induces autophagy in eukaryotic cells through inhibition of mTOR, a major negative regulator of autophagy [36]. mTOR can be phosphorylated (at serine 2448) by phosphorylated(p)-Akt-serine(S)473 to form p-mTOR-S2448 which inhibits the induction of autophagy [37]. mTOR positively regulates protein translation through the phosphorylation of its substrates, protein S6 Kinase (p70S6K), eukaryotic initiation factor 4E-binding protein 1 (4E-BP1) and S6 ribosomal protein (S6) [22]. In this study, to confirm whether Akt/mTOR pathway was involved in autophagy induced by asparaginase, we firstly evaluated the level of phosphorylated mTOR in asparaginase-treated K562 cells. Western blot analysis showed that asparaginase decreased the phosphorylation of mTOR in a dose- and time-dependent manner. Then we evaluated the expression of phosphorylation of Akt, an upstream inducer of mTOR. After dose- and time-dependently incubated with asparaginase, the level of phosphorylation of Akt significantly decreased. Furthermore, three downstream substrates of mTOR, p70S6K, 4E-BP1 and S6, showed significant decreases in phosphorylation (Figure 5A, 5B, 5C, and 5D).

Extracellular signal-regulated kinase (Erk1/2) has been shown to regulate expression of autophagy and lysosomal genes, and stimulate autophagy by interacting with LC3 [38, 39]. Recent studies have demonstrated new unconventional functions of autophagy (ATG) proteins and LC3-II in the upregulation of Erk phosphorylation [40]. In this study, an increased level of Erk1/2 phosphorylation (p-Erk1/2-T202/Y204) was observed in a dose- and time-dependent manner in K562 cells treated with different concentrations of asparaginase for 24 h (Figure 5E) or with 0.5 IU/mL of asparaginase for 3, 6, 12 and 24 h (Figure 5F). To further investigate the role of Erk1/2 in autophagy induced by asparaginase, U0126 (Erk phosphorylation inhibitor) was employed to block the phosphorylation of Erk1/2. Figure 5G revealed that the level of LC3-II as well as p-Erk1/2-T202/Y204 decreased in K562 cells after exposure to 0.5 IU/mL of asparaginase and 20 μM of U0126 for 24 h, indicating that autophagy was suppressed by inhibiting the phosphorylation of Erk.

These experiments suggest that the Akt/mTOR and Erk signaling pathway are involved in autophagy induced by asparaginase in K562 CML cells.

## DISCUSSION

CML is a myeloproliferative disease, which has high morbidity and mortality in human beings [1]. The TKIs are highly effective in CML treatment, while a problem that may arise due to the widespread use of TKIs is increased drug resistance [41]. Therefore, it is necessary to find novel therapeutic approaches to overcome this problem. The targeting of metabolic processes has revealed as a promising approach to cancer therapy. Asparaginase, a FDA-approved enzyme, is a cornerstone in the multi-drug treatment of childhood ALL and has been used for over 40 years [7, 42]. However, the anti-CML effect of asparaginase and its underlying mechanism has not been completely elucidated. In this study, we observed that asparaginase induced growth inhibition and apoptosis in K562 and KU812 cells. Further study illustrated that asparaginase-induced apoptosis was partially caspase 3-dependent in K562 cells, indicating one of the underlying mechanisms of anti-CML effect of asparaginase was the induction of apoptosis.

It has been well demonstrated that amino-acid depletion can induce autophagy [18, 21]. Previous research showed that L-asparaginase inhibited mTORC1 through its glutaminase activity and induced apoptosis as well as a strong autophagic process in AML cells [14]. Autophagy was also investigated in ovarian cancer cells upon asparaginase treatment [27]. In this study, we could not help asking whether asparaginase induced autophagy in CML cells? Three well-established methods were used to detect autophagosome formation. We observed asparaginase-induced autophagic response in K562 and KU812 cells as evidenced by the formation of autophagosome through TEM, LC3-positive autophagy-like vacuoles through Cyto-ID Green dye, and the increased conversion of LC3-I to LC3-II through western blot analysis.

Whether autophagy promotes cell death or enhances survival is still controversial [43, 44]. Although drug-induced autophagic tumor cell death has been reported [45–47], results from most studies support the survival role of autophagy in chemotherapy-induced cell death [19, 20, 25, 26]. The explanation for the complex process is thought to be specific to cell types, phases, genetic background and microenvironment [48]. What would be the role of autophagy in asparaginase-treated K562 and KU812 cells? To directly clarify this question, we inhibited asparaginase-induced autophagy pharmacologically by using LY294002, CQ and QN in K562 and KU812 cells. We found that asparaginase-induced cell death significantly increased by additional treatment with LY294002, CQ and QN. Moreover, microscope analysis showed that asparaginase in combination with LY294002, CQ or QN induced more obvious morphology changes including cell shrinkage, fragmentation, and death when compared with asparaginase-treated alone. Indicating asparaginase-induced autophagy might play a cytoprotective role in K562 and KU812 cells. To further confirm the cytoprotective role of autophagy induced by asparaginase in K562 and KU812 cells, we detected apoptosis in K562 and KU812 cells when cells were treated with asparaginase and autophagy inhibitors. Remarkably, LY294002, CQ and QN treatment enhanced asparaginase-induced apoptosis as evidenced by increased Annexin V-positive/PI-negative cells, caspase-3 cleavage, and PARP cleavage. All of these results demonstrated that asparaginase-induced autophagy played a cytoprotective role in K562 and KU812 cells. Blocking autophagy could enhance the efficacy of asparaginase on K562 and KU812 cells and this might be a promising new therapeutic strategy for CML. In our recent studies, arginase, another amnio acid-degrading enzyme, was found to induce apoptosis and cytoprotective autophagy in non-Hodgkin's lymphoma and melanoma, and inhibition of autophagy was demonstrated to enhance the antitumor effect of arginase [19, 20]. All our studies elucidated that autophagy induced by deprivation of essential amino acid played a cytoprotective role in malignant cancers, and inhibition of autophagy could enhance the antitumor efficacy of therapeutic enzyme.

Erk controls various aspects of cell physiology including autophagy. It has been shown that growth factor increases the interaction of Erk cascade components with autophagy proteins in both cytosol and nucleus [40]. Akt/mTOR signaling is another pathway to regulated autophagy. Akt negatively regulates autophagy via activation of mTOR, which inhibits multiple autophagy-promoting proteins via phosphorylation [26, 49]. In this study, we showed that upon asparaginase treatment the dose and time-dependent reduction of Akt and mTOR phosphorylation, as well as the phosphorylation substrates of mTOR (p-p70S6K-S371 and p-4EBP1-pT45 and p-S6-S235/S236) in K562 cells, indicating the Akt/mTOR signaling pathway was involved in asparaginase-induced autophagy in K562 cells. Whereas the same treatment showed increasement of Erk phosphorylation (p-Erk1/2-T202/Y204) through western blot analysis. We further confirmed the role of Erk pathway by using Erk phosphorylation inhibitor U0126. We found that inhibition of Erk phosphorylation downregulated the LC3 II level, thereby inhibiting autophagy. These results indicated that both Akt/mTOR and Erk signaling pathway were involved in autophagy induced by asparaginase in K562 CML cells.

Asparagine is required by all cells for survival and is normally produced by ASNS [8]. Asparaginase-sensitive malignant tumor cells are thought to express relatively low levels of ASNS and thus depend on the available of extracellular asparagine for their survival [9]. However, recent study showed that asparaginase exhibited significant cytotoxicity of ASNS-positive cancer cells including K562, SR leukemia cells, and this anticancer activity might due to the glutaminase activity of asparaginase [50].

**Scheme 1:**
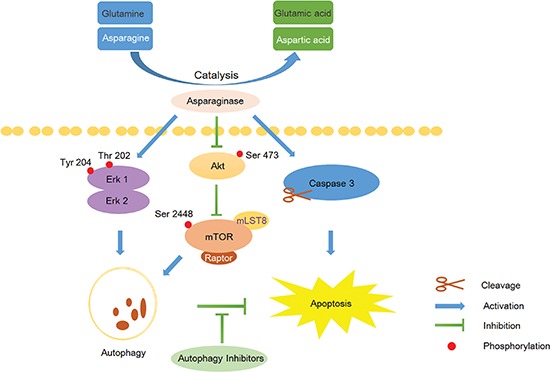
Overview of apoptosis and autophagy pathways induced by asparaginase in K562 CML cells Asparaginase catalyzes asparagine and glutamine to aspartic acid and glutamic acid respectively, and the resulting amino acid deficiency concurrently induces caspase 3-dependent apoptosis and autophagy in K562 cells. Moreover, the Akt/mTOR and Erk signaling pathway are involved asparaginase-induced autophagy in K562 cells. Inhibition of autophagy by the autophagy inhibitors can significantly enhance asparaginase-induced growth inhibition and cell apoptosis in K562 CML cells.

In conclusion, the present study proved that asparaginase could induce autophagy and apoptosis in K562 and KU812 CML cells, and autophagy induced by asparaginase played a cytoprotective role. Inhibition of autophagy by the autophagy inhibitors LY294002, CQ and QN could significantly enhance growth inhibition and cell apoptosis in K562 and KU812 cells. Furthermore, our results suggested that the Akt/mTOR and Erk pathway were involved in asparaginase-induced autophagy in K562 cells (Scheme 1). Our research highlighted that combination of asparaginase and autophagic inhibition might be a promising new therapeutic strategy for CML.

## MATERIALS AND METHODS

### Materials and buffers

Asparaginase (derived from *Erwinia*) was purchased from Baiyunshan Mingxing Pharmaceutical Co., Ltd. (Guangzhou, Guangdong Province, China). Both of the autophagy inhibitors, the PI3K inhibitor LY294002 and the lysosomal inhibitor CQ, were obtained from Sigma-Aldrich (St Louis, MO, USA). Another autophagy inhibitor QN was purchased from Aladdin Industrial Corporation (Shanghai, China) The autophagy inducer Rapamycin was purchased from Beyotime Institute of Biotechnology (Haimen, Jiangsu Province, China). The caspase inhibitor z-VAD-fmk was obtained from Beyotime Institute of Biotechnology (Haimen, Jiangsu Province, China). Fluorescein (FITC)-Annexin V Apoptosis Detection kit was purchased from BD Bioscince (Franklin Lakes, NJ, USA). 3-(4,5-dimetrylthiazol-2-yl)-2,5-diphenyltetrazolium bromide (MTT) was purchased from Sigma-Aldrich (St. Louis, MO, USA). U0126, a MEK1/2 inhibitor, was obtained from Cell Signaling Technology (Danvers, MA, USA). The antibodies including anti-β-actin, anti-Tubulin, anti-cyclin D, anti-LC3B, anti-caspase 3, anti-cleaved caspase 3, anti-PARP, anti-cleaved PARP, anti-phospho-mTOR (Ser2448), anti-mTOR, anti-phospho-Akt (Ser473), anti-Akt, anti-p70S6 Kinase Phospho (pS371), anti-phospho-S6 (Ser235/236), anti-phospho-4EBP1-pT45, anti-phospho-p44/42 MAPK (Erk1/2) (Thr202/Tyr204) and anti-p44/42 MAPK (Erk1/2) were obtained from Cell Signaling Technology (Danvers, MA, USA). The secondary antibodies horseradish peroxidases (HRP)-conjugated goat anti-mouse and anti-rabbit immunoglobulin G were purchased from MR Biotech (Shanghai, China).

### Cell culture

Human CML cell line K562 and KU812 were purchased from Cell Bank of Chinese Academy of Sciences, Shanghai Branch (Shanghai, China). K562 cells were cultured in RPMI-1640 containing 10% of heat-inactivated fetal bovine serum (FBS), and KU812 cells were maintained in IMDM medium with 15% FBS. All the medium were containing 100 U/mL of penicillin and 100 μg/mL of streptomycin. The cells were grown at 37°C in a 5% CO2 atmosphere incubator.

### Cell viability assay

Cell viability was measured by the MTT cytotoxicity assay. About 1 × 104 cells were seeded in 96-well plates and then incubated with different dilutions of asparaginase with or without autophagy inhibitors. After treatment for 48 h, cells were incubated with 0.5 mg/mL of MTT for 4 h at 37°C. Then, 100 mL of 20% SDS in dimethyl formamide/H2O (1 : 1, v/v; pH 4.7) was added to each well, and dissolved formazan to solution for measurement. The optical density (OD) was measured at an absorbance wavelength of 570 nm.

### Western blot analysis

For western blot, K562 and KU812 cells were harvested and washed with cold phosphate-buffered saline (PBS). The proteins were extracted with RIPA Cell Lysis Buffer (Beyotime Institute of Biotechnology, Haimen, China), and kept on ice for at least 30 min. The lysates were centrifuged at 12,000g at 4°C for 10 min, then the supernatant was transferred to a fresh tube. After protein concentration was measured by the bicinchoninic acid (BCA) method, an equal quantity of total protein per lane was separated by sodium dodecyl sulfate-polyacrylamide gel electrophoresis (SDS-PAGE) and transferred to polyvinylidene fluoride (PVDF) membranes. Membranes were blocked with 3% bovine serum albumin (BSA) powder in 0.05% Tris-buffered saline and Tween 20 (TBST) for 1 h at room temperature and then incubated overnight at 4°C with specialized antibodies. After overnight incubation, membranes were washed for three times and then incubated for 2 h at room temperature with peroxidase-conjugated secondary antibodies. Detection was performed with enhanced chemiluminescence reagents (Pierce, Rockford, IL, USA). Intensities in the resulting bands were quantified by IQuantTL software (GE Healthcare, USA).

### Apoptosis assay

Annexin V-FITC/PI Detection Kit (BD Biosciences, San Diego, CA, USA) and Annexin V-FITC/PE Detection Kit (Beyotime Institute of Biotechnology, Haimen, Jiangsu Province, China) were used for the determination of cell apoptosis. K562 and KU812 cells were exposed to asparaginase with or without autophagy inhibitors for 48 h, then harvested and washed twice with cold PBS, and re-suspended in 1× binding buffer at a concentration of 1 × 10^6^ cells/mL. Subsequently, according to the manufacturer's instructions, the cells were stained with annexin V-FITC and PI/PE for 15 min at 37°C. Then, the cells were analyzed immediately by using a FACS Calibur flow cytometer (Becton-Dickinson, Fullerton, CA, USA).

### Cell cycle analysis

The effect of asparaginase on K562 cell cycle distribution was determined by FACS Calibur flow cytometer (Becton-Dickinson, Fullerton, CA, USA) analysis. After incubation with 0.02, 0.1, and 0.5 IU/mL of asparaginase for 48 h, K562 cells were fixed in 70% ethanol at the temperature of −20°C for overnight, washed twice with cold PBS, and stained with PI and RNaseA at 4°C for 30 min. Then, the samples were analyzed by FACS Calibur flow cytometer.

### Transmission electron microscopy analysis

TEM assays were performed as described in our previous study [25]. K562 and KU812 cells were incubated with 0.5 IU/mL of asparaginase for 24 h, then harvested and fixed with ice-cold glutaraldehyde. Samples were detected with a JEM 1410 transmission electron microscope (JEOL, Inc., USA) at 80 kV.

### Microscopy and photography

About 1 × 104 K562 and KU812 cells were seeded into 96-well plates and then incubated with different dilutions of asparaginase with or without autophagy inhibitors. After incubation for 48 h, cells were examined by using an inverted microscope (Nikon, Japan) equipped with a model digital camera.

### Confocal microscopy

K562 and KU812 cells were seeded into 6-well plates at a density of 1 × 105/mL and then treated with 0.5 IU/mL of asparaginase. After 24 h of incubation, cells were stained with Cyto-ID® Green dye and Hoechst 33342 at 37°C for 30 min according to the manufacturer's protocol. Then the cells were washed and re-suspended with PBS. A drop of the cell suspension were taken to a glass microscope slide and overlaid with a coverslip and immediately analyzed by confocal microscopy. Positive controls were treated with the autophagy inducer Rapamycin at 50 nM for 12 h, and disposed with same steps. All the procedures were done in the dark place.

### Statistical analysis

Data from this study were presented as mean values with standard deviations (SD). The statistical significance of the differences between groups was evaluated by Student t test. *, **, and *** indicated *P* < 0.05, *P* < 0.01 and *P* < 0.001, respectively.

## SUPPLEMENTARY FIGURES


