# Supplementary material for: Asparaginase induces apoptosis and cytoprotective autophagy in chronic myeloid leukemia cells
Source: Oncotarget. 2015 Jan 8;6(6):3861–73. doi: 10.18632/oncotarget.2869 (PMC4414159; doi:10.18632/oncotarget.2869)
Supplement: Supplementary file 1 [file oncotarget-06-3861-s001.pdf]

SUPPLEMENTARY FIGURES

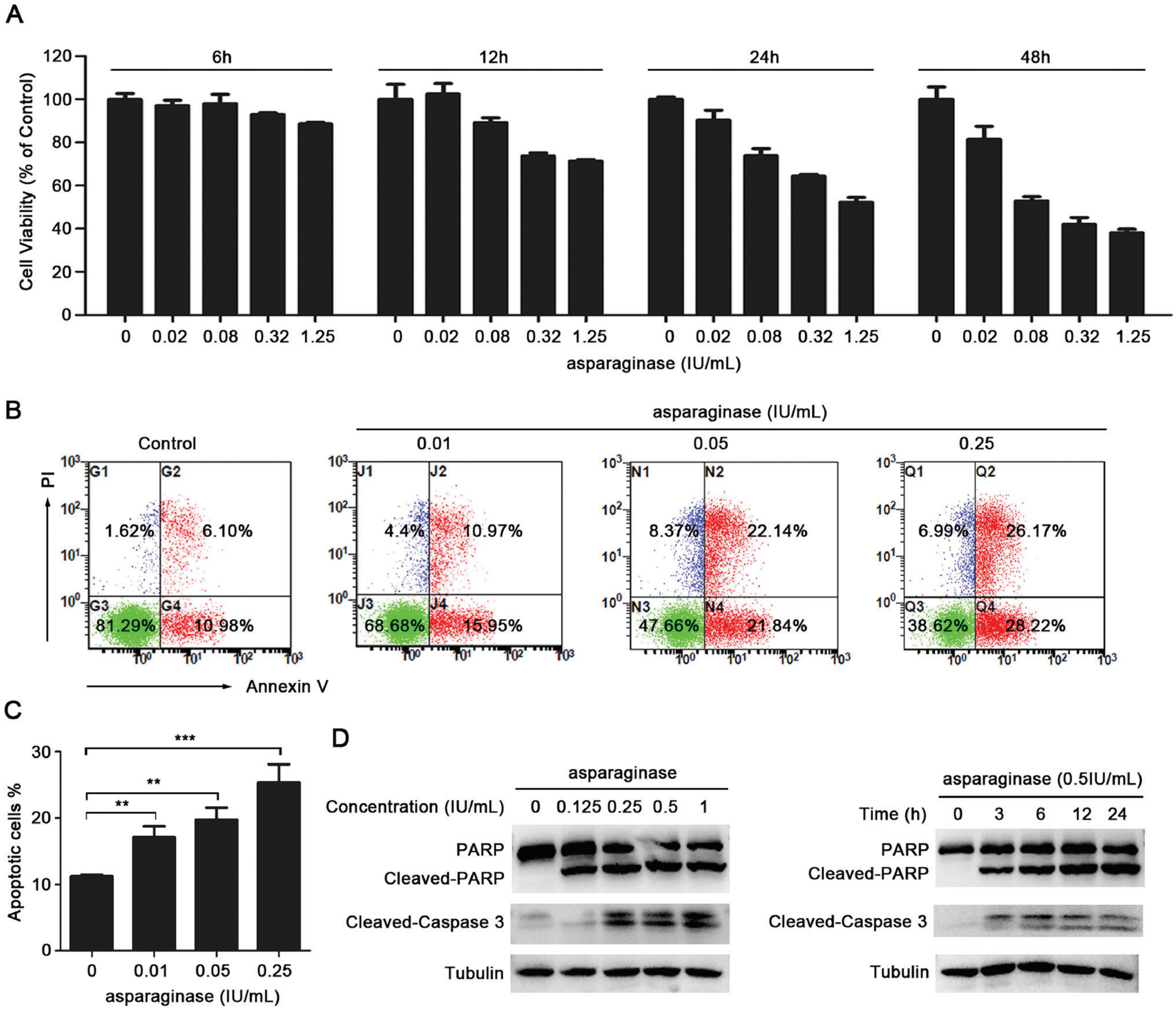

Supplementary Figure 1: Asparaginase induces growth inhibition and apoptosis in KU812 CML cells. (A) KU812 cells were incubated with different concentrations of asparaginase for 6, 12, 24, and 48 h, and then cell viability was measured by MTT assay. **(B)** KU812 cells were treated with 0.01, 0.05, 0.25 IU/mL of asparaginase for 48 h, and stained with Annexin V/PI, then analyzed by flow cytometry. **(C)** The percentages of Annexin V-positive/PI-negative cells were presented in bar charts. **(D)** KU812 cells were dose- and time-dependently treated with asparaginase, then western blot analysis was performed to assess the expression level of cleaved-caspase 3, PARP, and cleaved-PARP. Results were represented as mean ± SD (**$P < 0.01$, ***$P < 0.001$).

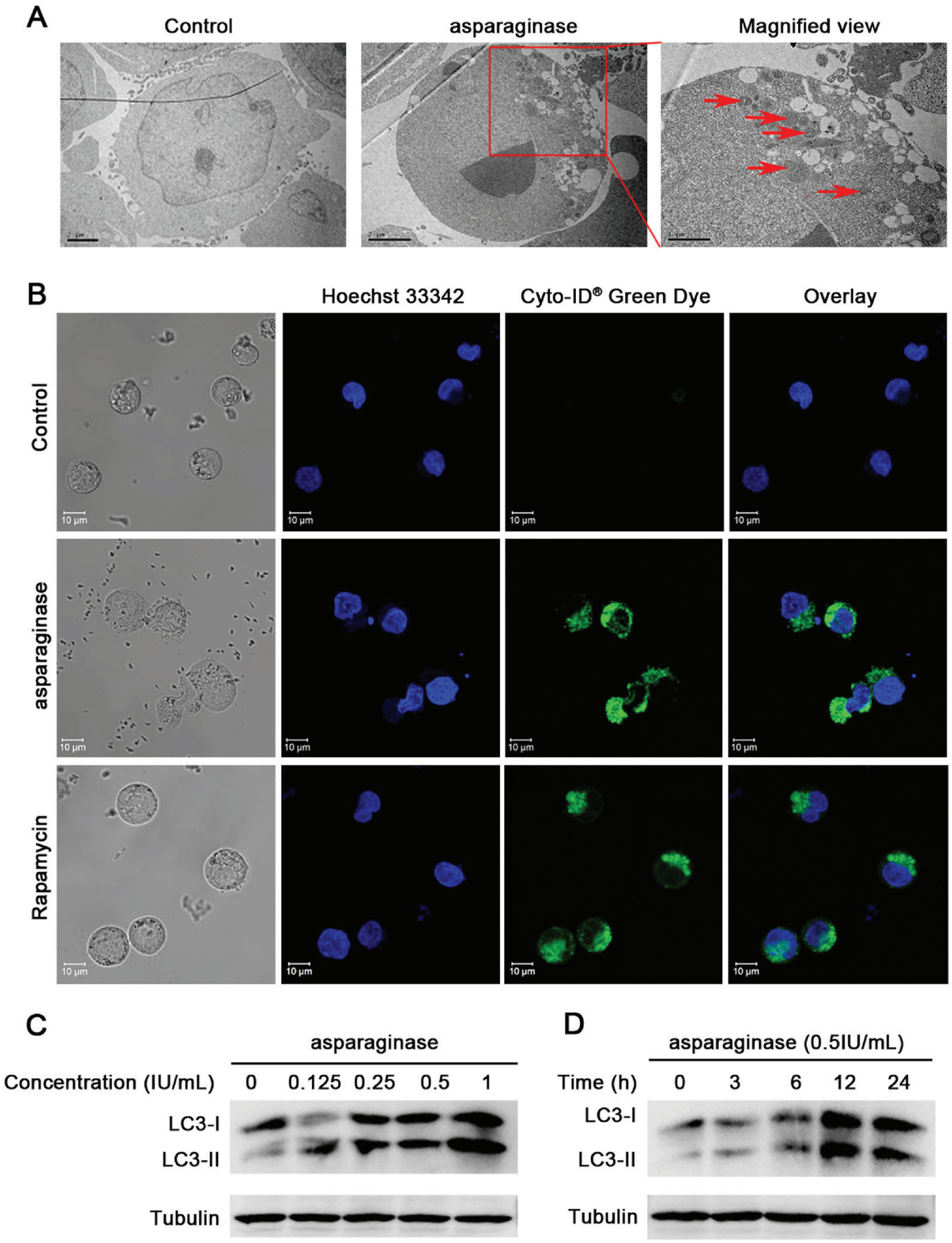

Supplementary Figure 2: Autophagy is induced by asparaginase in KU812 cells. (A) KU812 cells were treated with 0.5 IU/mL of asparaginase for 24 h. TEM was employed to detect the autophagosomes ("red arrows": autophagosomes). **(B)** KU812 cells were treated with 0.5 IU/mL of asparaginase for 24 h, then cells were stained with Cyto-ID® Green autophagy dye and examined by confocal fluorescent microscopy. **(C)** KU812 cells were treated with 0.125, 0.25, 0.5 and 1 IU/mL of asparaginase for 24 h, then detected autophagy-associate protein LC3-I/II by western blot analysis. **(D)** KU812 cells were treated with 0.5 IU/mL of asparaginase for 3, 6, 12 and 24 h, the expression level of LC3-I/II were evaluated by western blot analysis.

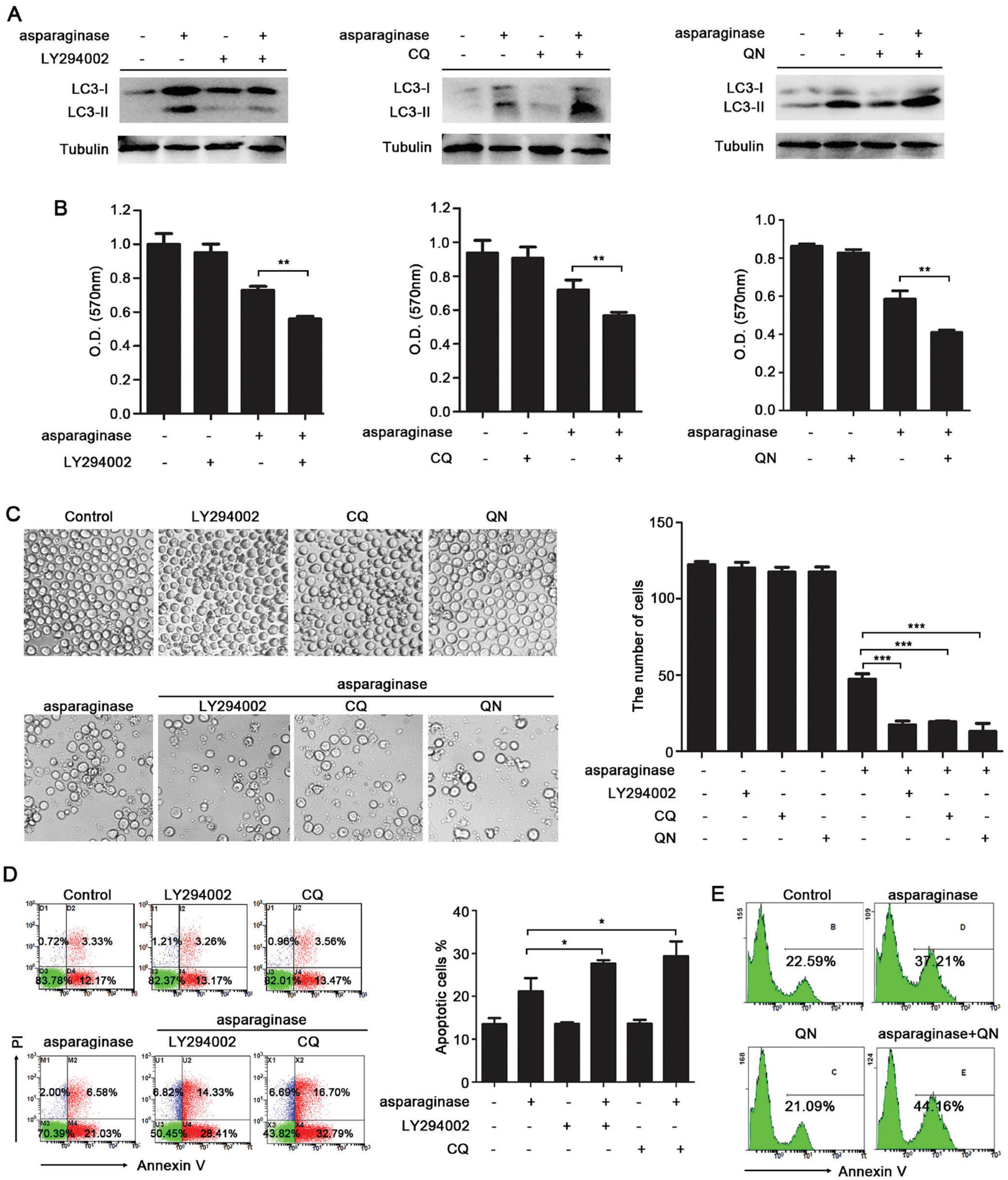

Supplementary Figure 3: Inhibition of autophagy enhances asparaginase-induced KU812 cell death. **(A)** KU812 cells were treated with 0.02 IU/mL of asparaginase in the absence or presence of 5 µM LY294002, 5µM CQ or 1µM QN for 24 h, autophagy-associated protein LC3-I/II were detected by western blot analysis. **(B–C)** KU812 cells were incubated with 0.02 IU/mL of asparaginase in the absence or presence of 5 µM LY294002, 5 µ M CQ or 1 µ M QN for 48 h. **(B)** Cell viability was analyzed by MTT assay. **(C)** Morphological and numerary changes of KU812 cells were observed using microscopy and photography. The number of normal cells was presented in bar charts. **(D)** KU812 cells were incubated with 0.02 IU/mL of asparaginase in the absence or presence of 5 µM LY294002 or 5 µM CQ, cell apoptosis was detected by Annexin V-FITC/PI staining. The percentage of Annexin V-positive/PI-negative KU812 cells was presented in bar charts. **(E)** KU812 cells were incubated with 0.02 IU/mL of asparaginase in the absence or presence of 1 µM QN, cell apoptosis was detected by Annexin V-FITC/PE staining.

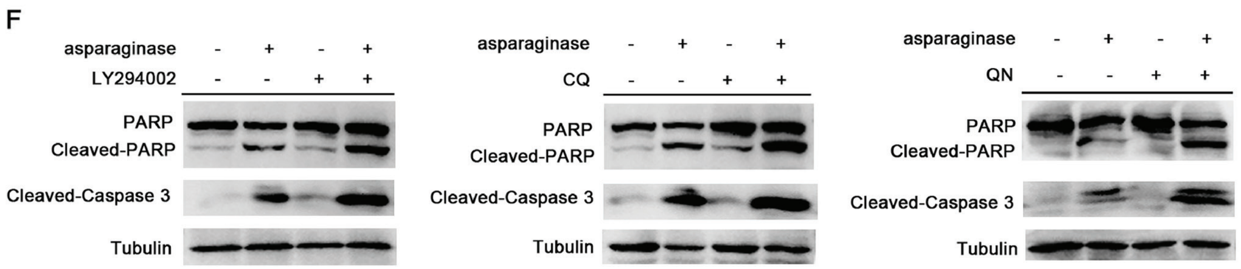

Supplementary Figure 3: (*Continued*) (F) KU812 cells were treated with 0.02 IU/mL of asparaginase in combination with or without 5 μM LY294002, 5 μM CQ or 1μM QN for 24 h, the expression level of protein cleaved-caspase 3, PARP and cleaved-PARP were analyzed by western blot analysis. Results were represented as mean ± SD (*P < 0.05, **P < 0.01, ***P < 0.001).

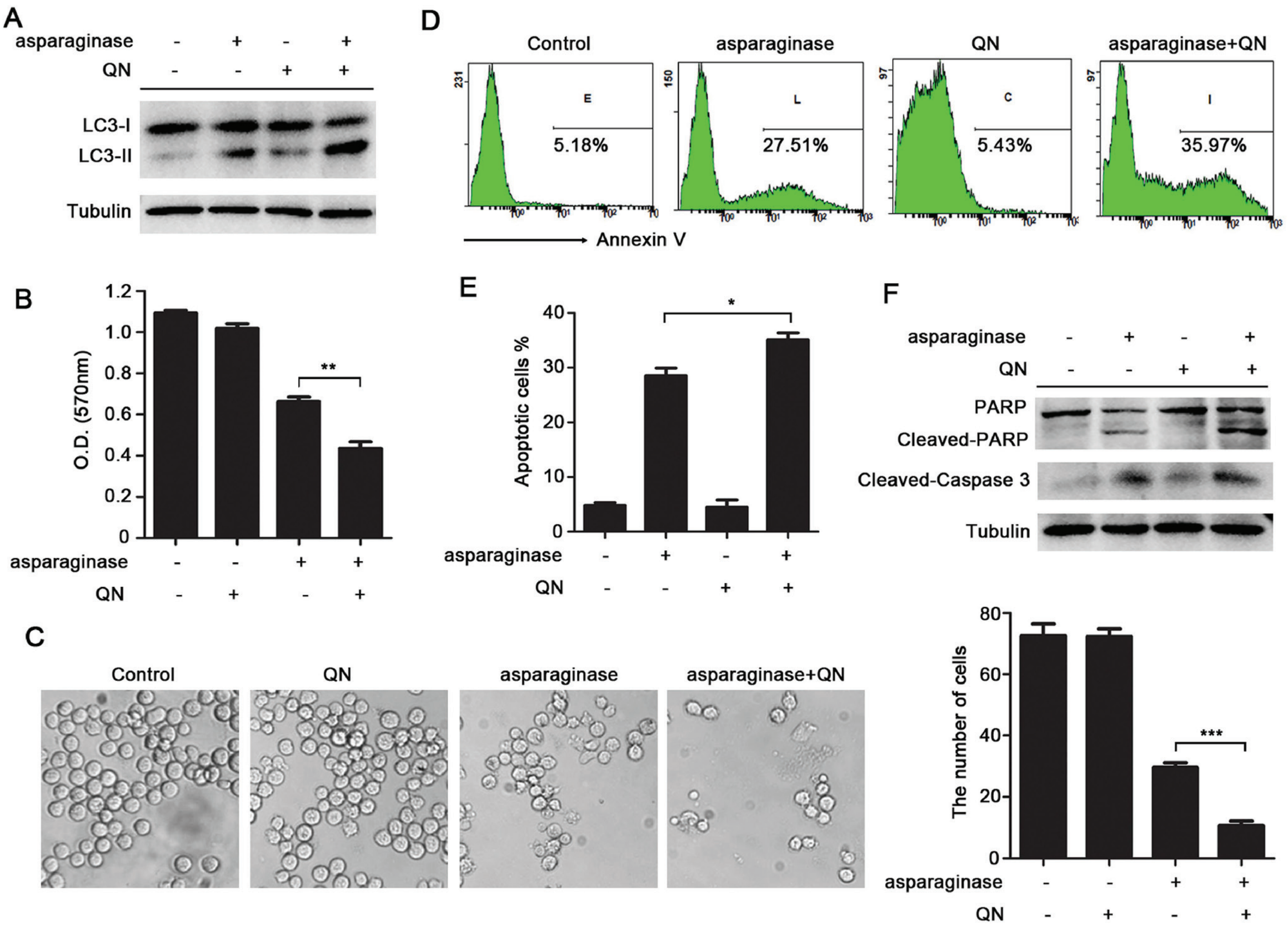

Supplementary Figure 4: Inhibition of autophagy enhances asparaginase-induced K562 cell death. **(A)** K562 cells were treated with 0.04 IU/mL of asparaginase in the absence or presence of 1.5 µM QN for 24 h, autophagy-associated protein LC3-I/II were detected by western blot analysis. **(B–E)** K562 cells were incubated with 0.04 IU/mL of asparaginase in the absence or presence of 1.5 µ M QN for 48 h. **(B)** Cell viability was analyzed by MTT assay. **(C)** Morphological and numerary changes of K562 cells were observed using microscopy and photography. The number of normal cells was presented in bar charts. **(D)** Cell apoptosis was detected by Annexin V-FITC/PE staining. **(E)** The percentage of apoptotic cells was presented in bar charts. **(F)** K562 cells were treated with 0.04 IU/mL of asparaginase in combination with or without 1.5 µ M QN for 24 h, the expression level of protein cleaved-caspase 3, PARP and cleaved-PARP were analyzed by western blot analysis. Results were represented as mean ± SD (**P* < 0.05, ***P* < 0.01, ****P* < 0.001).
